# Feedback control over plasma drug concentrations achieves rapid and accurate control over solid-tissue drug concentrations

**DOI:** 10.21203/rs.3.rs-5868915/v1

**Published:** 2025-01-28

**Authors:** Nicole Emmons, Zeki Duman, Murat Erdal, Tod Kippin, Joao Hespanha, Kevin Plaxco

**Affiliations:** University of California, Santa Barbara; University of California, Santa Barbara; University of California, Santa Barbara; University of California, Santa Barbara; University of California, Santa Barbara; University of California, Santa Barbara

## Abstract

Electrochemical aptamer-based (EAB) sensors enable the continuous, real-time monitoring of drugs and biomarkers in situ in the blood, brain, and peripheral tissues of live subjects. The real-time concentration information produced by these sensors provides unique opportunities to perform closed-loop, feedback-controlled drug delivery, by which the plasma concentration of a drug can be held constant or made to follow a specific, time-varying profile. Motivated by the observation that the site of action of many drugs is the solid tissues and not the blood, here we experimentally confirm that maintaining constant plasma drug concentrations also produces constant concentrations in the interstitial fluid (ISF). Using an intravenous EAB sensor we performed feedback control over the concentration of doxorubicin, an anthracycline chemotherapeutic, in the plasma of live rats. Using a second sensor placed in the subcutaneous space, we find drug concentrations in the ISF rapidly (30–60 min) match and then accurately (RMS deviation of 8–21%) remain at the feedback-controlled plasma concentration, validating the use of feedback-controlled plasma drug concentrations to control drug concentrations in the solid tissues that are the site of drug action. We expanded to pairs of sensors in the ISF, the outputs of the individual sensors track one another with good precision (*R*^*2*^ = 0.95–0.99), confirming that the performance of in vivo EAB sensors matches that of prior, in vitro validation studies. These observations suggest EAB sensors could prove a powerful new approach to the high-precision personalization of drug dosing.

## Introduction

**Figure 1:**
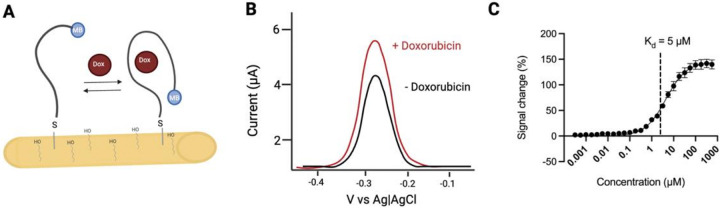
**(A**) Electrochemical aptamer-based (EAB) sensors are composed of a methylene blue (MB)-modified aptamer that is site-specifically attached to the surface of a gold electrode. Target binding produces a conformational change in this aptamer, altering the rate of electron transfer. **(B)** The binding-induced change in electron transfer results in an easily detectable change in peak current when the sensor is interrogated using square-wave voltammetry. **(C)** To perform measurements of doxorubicin in plasma and subcutaneous space, we utilize a doxorubicin aptamer with a K_d_ of 5 μM. Data was collected in vitro in whole rat blood at 37°C, with the error bars shown on this graph reflecting standard deviations across 8 independently fabricated devices to illustrate reproducibility. Figure created in BioRender.

Electrochemical aptamer-based (EAB) sensors enable real-time, seconds-resolved measurements of the disposition of drugs and biomarkers in the living body (Fig. 1A, 1B). They have been used, for example, to measure an increasing number of drugs and metabolites in situ in the blood (jugular),^1–4^ cerebrospinal fluid (lateral ventricle, hippocampus),^5^ and interstitial fluid (subcutaneous space, muscle, solid tumors).^6,7,8,9^ The real-time, in vivo concentration measurements provided by EAB sensors also offer unique opportunities to perform feedback-controlled drug delivery. We have previously demonstrated, for example, feedback control over both plasma^10,11^ and in-brain^5^ drug concentrations, in each case precisely achieving either fixed concentrations or pre-defined, time-varying concentration profiles with the latter, for example, mimicking human pharmacokinetics in a rat. Here we expand on these studies by using feedback control over plasma drug levels to explore two questions regarding the measurement of and control over drug concentrations in the solid peripheral tissues. The first question, which is clinical in focus, is how well and how rapidly control over plasma drug concentrations equates to control over drug concentrations in these tissues? The second question, which is technical in nature, is how accurate is the calibration of subcutaneous EAB sensors?

To motivate the clinical question we address, we note that the argument in favor of performing feedback control over plasma drug concentrations is that, if this is performed for long enough, drug concentrations in the solid tissues will eventually reach and maintain the same level. That is, the assumption that holding plasma drug concentrations constant at their clinically optimal level equates to achieving similarly constant, similarly optimal levels in the solid tissues that are the site of action of most drugs. Such an advance could prove particularly important in the pharmacological treatment of cancer, which generally assumes that achieving adequate plasma drug exposure corresponds to achieving adequate exposure in the solid tissues of a tumor. Here we test this assumption by employing feedback control to maintain a constant drug concentration in the plasma while simultaneously observing the resulting concentration in interstitial fluid (ISF) of the subcutaneous space.

To motivate the technical question we wish to address, we note that the calibration of sensors deployed in ISF has historically proven problematic.^12,13,14^ As an example, we discuss the situation with in vivo EAB sensors. We have previously calibrated such sensors in vitro in the physiological medium in which they will be deployed, such as undiluted whole blood for intravenous sensors^1,2,4,15^ and undiluted cerebrospinal fluid for intracranial sensors^5^, both held at body temperature. For sensors deployed in solid tissues, however, selecting the appropriate calibration medium is complicated by the difficulty of meaningfully collecting ISF. That is, ISF extraction methods, including suction blisters, microdialysis, and microneedle extraction, are limited to small volumes (~ 1–15 μL) and likely alter the ISF composition (e.g., by rupturing cells or dilution with dialysate) in the collection process.^13,16–19^ Given these limitations, some groups have entirely eschewed the calibration of subcutaneous EAB sensors, reporting instead relative signal change (in percent) rather than estimated concentrations.^20^ Here, in contrast, we have calibrated subcutaneous EAB sensors using 37°C whole blood as the calibrant, an approach we justified by observing that, while they are filled with a molecular-weight-limiting “glycocalyx” matrix^21,22^ the junctions between the cells lining the walls of the finest capillaries are “leaky” enough that there is pressure-driven bulk flow between the plasma and ISF,^13,23^ suggesting that the electrolyte and small-molecule composition of the two should be closely similar. Consistent with this argument, 90% of the metabolites found in the ISF are also found in blood,^13,24–26^ suggesting that the electrolyte and small-molecule composition of the two should be closely similar. By coupling feedback control over plasma drug levels with simultaneous measurements performed in the ISF, we can quantitatively test the accuracy of using blood as a proxy calibrant for the latter. Our argument is that, under these conditions. the estimated concentration in the ISF should eventually match and remain at the plasma concentration provided that blood is an accurate calibration matrix for sensors deployed in the ISF.

## Results

**Figure 2:**
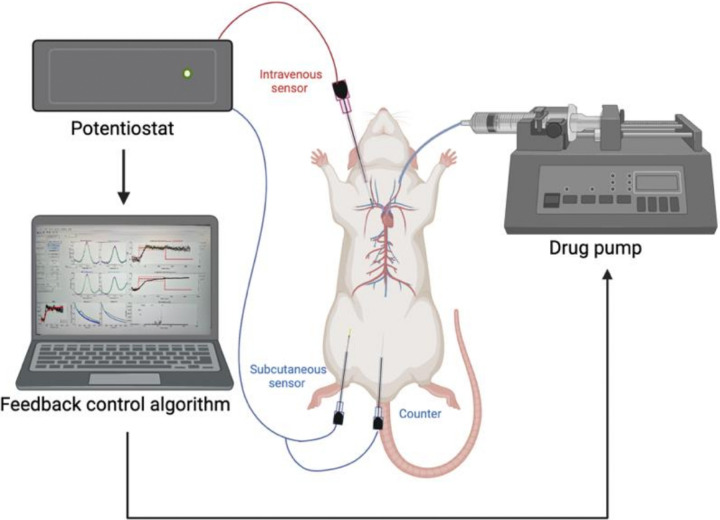
To perform feedback-control over plasma drug concentrations, we insert an intravenous sensor in the right jugular vein and an indwelling catheter into the left jugular vein and then connect it to a potentiostat for electrochemical interrogation using square-wave voltammetry. The real-time data this produces is used to inform an adaptive feedback control algorithm ^33^ that adjusts the rate with which a drug pump delivers the drug to rapidly reach and accurately maintain the desired set point. Here we also employed 1 or 2 sensors in the ventral subcutaneous space to measure how rapidly these solid tissues equilibrate with the plasma drug concentration. Figure created in BioRender.

Given that feedback-controlled drug delivery will presumably be of the greatest value for drugs for which the pharmacokinetic variability between patients is clinically significant,^27,28^ we have used the chemotherapeutic doxorubicin, a drug whose high inter- and even intra-subject variability is known to impact clinical outcomes significantly.^29,30^ To measure the plasma doxorubicin concentrations we placed a 200 μm diameter by 3 mm long EAB sensor into the right external jugular vein^31^ of anesthetized rats (subject characteristics detailed in Table S1) and a catheter into the left jugular vein for drug delivery. To perform similar measurements in the ISF, we inserted 200 μm diameter by 5 mm long EAB sensors into the subcutaneous space by first inserting 18-gauge shielded catheters below the surface of the skin on the animal’s posterior ventral side at a 45° angle. We then removed the needles, leaving the catheters in place to guide the sensors into the subcutaneous tissue (Fig. 2). To interrogate both intravenous and subcutaneous sensors we used square-wave voltammetry to perform dual-frequency, kinetic differential measurements (KDM).^7,15^ Using this approach, our sensors achieve a time resolution of 16 s and baseline (pre-drug-challenge), root-mean-squared noise floors of 0.4 μM in the vein, and 0.2 μM in the subcutaneous space. The greater noise in the former compartment arises due to the animal’s pulse; this noise falls dramatically upon sacrificing the animal (data not shown). To maintain constant drug concentrations in the plasma we employed an adaptive feedback control algorithm that actively “learns” the pharmacokinetics of each animal.^32^ Relative to our earlier, proportional-integral-derivative controllers^5,10^ this approach improves the speed with which the set point concentration is reached and the accuracy with which this is maintained.

**Figure 3:**
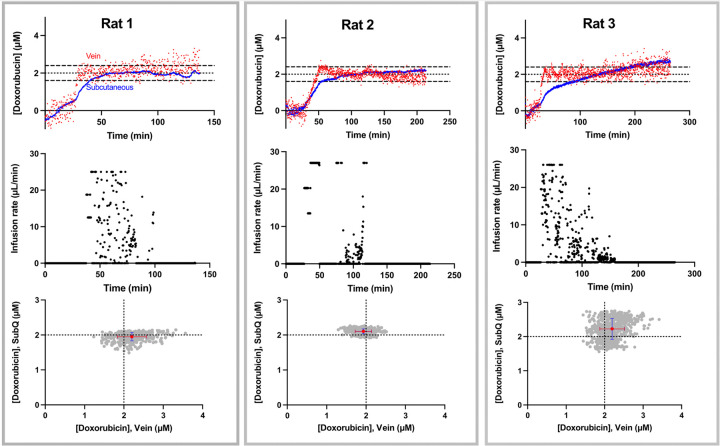
After the initiation of feedback control, drug levels in the subcutaneous ISF rapidly equilibrate with those in the plasma. (Top Row) Shown are data sets collected from three different animals (the three columns) illustrating the measured concentration in the plasma (red) and in the ISF (blue). In all three cases the drug concentration in the plasma and ISF rapidly rise to the set point. (Middle Row) Shown are the time-varying infusion rates required to reach and maintain the set-point plasma concentration. Note that the maximum infusion rate differs between rats depending on their body weight. (Bottom Row): After drug concentrations in the plasma and ISF have equilibrated (defined here as subcutaneous concentrations reaching and remaining within 15% of the set point for at least 5 min), the two concentrations remain closely similar. To illustrate this, here we present scatter plots illustrating the correlations between the pairs of measured concentrations after equilibration has been reached. The red points represent the average of vein and subcutaneous measurements obtained over this period, and the error bars reflect the standard deviations of each value. The generally larger standard deviations seen for plasma measurements are due to noise arising from the close placement of these sensors to the animal’s heart. Note: we hypothesize that the slight upward drift seen for both the intravenous and subcutaneous measurements in rat 3 may be due to continued, slow leaking of drug from the pump, as there is no valve that separates it from the animal.

**Figure 4:**
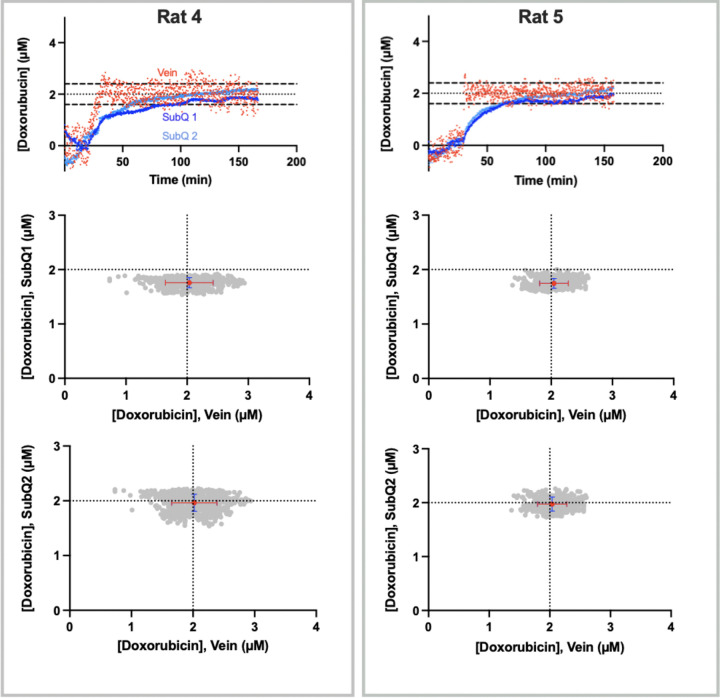
We observe good reproducibility between pairs of subcutaneous sensors placed within individual animals. (Top row) To see this, here we deployed a pair of sensors in the ventral subcutaneous space located ~1 cm away from one another. (Bottom rows) Once again, after the subcutaneous space has equilibrated with the plasma (determined when the subcutaneous levels reach within 15% of the target concentration and maintain set point concentration for at least 5 min), the correspondence between the measured plasma and ISF drug concentrations is excellent for both sensors in the pair. Red points represent the average vein and subcutaneous concentration across the duration of the experiment following equilibration. Error bars represent the standard deviation of the in-vein measurements (red) and the subcutaneous measurements (blue). The corresponding infusion rate data is presented in the SI (Fig. S2).

Feedback control over plasma drug levels leads to rapid equilibration at the same concentration in the interstitial fluid of the subcutaneous space. To see this, in our first experiment we placed an intravenous sensor in the right external jugulars of 3 rats and a second sensor in the subcutaneous space of the right posterior ventral side. After establishing a stable baseline for the plasma sensor (typically after 20 to 30 min, Table 1), we initiated controller-informed drug infusion (Fig. 3, top row). Under such control, the plasma doxorubicin rose to the 2 μM set point (within 15%) in 2 to 11 mins (average of 6.8 min; Table 1), after which the controller maintained the plasma concentration at that set point to an RMS deviation of ± 30% (Fig. 4). Of note, the rate of infusion required to maintain the set point concentration, which is evident in infusion rate profiles (Fig. 3, middle row), varied not only between animals but also within individual animals over time. Specifically, the infusion rate required to maintain plasma drug concentrations at the set point concentration eventually dropped to 0 in all 3 animals. This is not due to poor sensor reversibility; the sensor rapidly returns to baseline after being transferred to 37°C, doxorubicin-free blood in vitro (Fig. S2). Instead, we believe this is due to doxorubicin-induced kidney failure reducing the drug’s elimination rate to zero.^34,35^ Consistent with this argument, all 3 rats stopped urinating during the experiment despite the significant intravenous fluid they received as part of the drug delivery process.

Controlling plasma drug concentrations equates to control over drug concentrations in the solid tissue. Specifically, after the ISF has equilibrated with the plasma, (defined here as being when the subcutaneous concentration reaches within 15% of the plasma set point concentration and remains within this window for at least 5 min), the mean measured plasma concentration and the mean measured ISF concentration differ by between 0.10 and 0.29 μM for our first 3 animals (Fig. 3, bottom row, Table 2). In the face of sensor-to-sensor fabrication variation, which is known to be of similar magnitude for these crudely hand-built devices,^15^ this rather high degree of correlation validates both the argument that control over plasma drug concentrations leads to rapid and accurate control over drug concentrations in the solid peripheral tissues and our use of blood as a calibrant for sensors deployed in the ISF.

**Figure 5:**
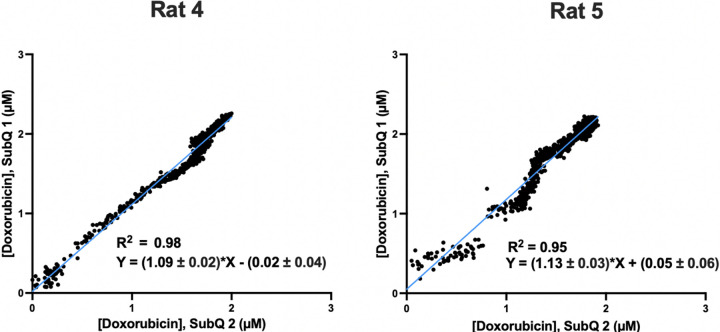
Measurements performed simultaneously at two sites in the subcutaneous space track one another with good precision. Shown are simple linear regressions of the concentration estimates produced by each of the paired subcutaneous sensors presented in Fig. 4; the Pearson correlations between the paired measurements are exceptional: R^2^ = 0.98, F(1, 762) = 33167, p < 0.0001 and R^2^ = 0.95, F(1, 905) = 16472, p < 0.0001) for rats 4 and 5, respectively. This said, we observe mean, systematic deviations between the two sensors of 9±2% (Rat 4) and 13±3% (Rat 5). This presumably occurs due sensor-to-sensor fabrication variation; equivalent levels of deviation have been reported during in the in vitro characterization of similarly hand-made devices.^15^

The reproducibility of subcutaneous EAB sensors is quite good. To see this, we next performed feedback control over plasma levels in 2 rats while performing concentration measurements using a pair of subcutaneous sensors placed ~ 1 cm apart, roughly 0.5 cm to either side of the animal’s midline. Once again, upon initiating feedback control over plasma doxorubicin, the drug’s measured concentration at both subcutaneous sites rapidly rises to and maintains the set point concentration (Fig. 4). Despite the possibility of site-specific differences in the rate of transfer from the plasma to the ISF, the concentration measurements produced by the two subcutaneous track one another quite closely. Specifically, measurements following the initiation of feedback control are correlated between the two sensors with *R*^*2*^ of 0.95 and 0.98 (Fig. 5). That said, the slopes of these correlations differ from the expected unity by 9% and 13%. These small, systematic errors presumably arise due to sensor-to-sensor variation in these hand-built devices. For example (and as noted above), we observe similar magnitude variations when hand-built EAB sensors are challenged in vitro in 37° C whole blood.^15^

## Discussion

Here we have performed feedback control over plasma drug concentrations in live rats with simultaneous monitoring of the drug in a solid tissue. Doing so we find that, upon holding plasma doxorubicin concentrations steady, the measured concentration in the interstitial fluid of the subcutaneous space matches that seen in plasma to within ~ 15%, a level of concordance well within expected sensor-to-sensor variability. And it achieves this concordance in a few tens of minutes, a timescale quite rapid relative to the action of most drugs. The effectively equivalent concentrations in the two compartments suggest that transport between the two occurs via passive diffusion. In contrast, were active transport occurring, which would create and maintain a concentration gradient, we would not expect convergence at equivalent concentrations.

From the technical perspective, the close correspondence of paired plasma and ISF drug concentration measurements following the equilibration of the ISF strongly supports our prior argument that blood is a suitable proxy for the calibration of sensors placed in the subcutaneous space. Given the difficulty of collecting sufficient volumes of undiluted, unperturbed ISF, this observation should significantly improve the convenience of future efforts to calibrate sensors for such placements. Indeed, given that most solid tissues (the exceptions being the brain and gonads^36,37^) are in direct, fluid communication with the plasma (i.e., are not separated by a transport-limiting membrane, such as the blood-brain-barrier), we believe this approach will work for a wide range of solid-tissue EAB sensor placements.

From the clinical perspective, the relatively rapid rate with which drug concentrations in the subcutaneous ISF equilibrate with constant plasma drug concentrations speaks to the potential therapeutic value of performing feedback control over plasma drug levels. Specifically, our work validates the assumption that holding drug concentrations constant at clinically optimized levels equates to achieving similarly optimal concentrations in the solid tissues that are the site of action of most drugs. This would advance the therapeutic administration of drugs with high interpatient pharmacokinetic variability and narrow therapeutic windows, a group of drugs that includes many chemotherapeutics.

## Materials and Methods

We diluted phosphate-buffered saline (PBS) from a 20x stock obtained from Santa Cruz Biotechnologies. Sodium hydroxide, 6-mercapto-1-hexanol, tris(2-carboxyethyl) phosphine, and used sulfuric acid (Sigma Aldrich) doxorubicin HCl (LC Laboratories) and dimethyl sulfoxide (Fisher Scientific) as obtained. We purchased methylene blue-and-HO-C 6 S-S-C 6-modified DNA sequences^3,20^ from Integrated DNA Technologies (IDT).

### Doxorubicin aptamer sequence

5’ – HS- (CH_2_)_6_ – ACCATCTGTGTAAGGGGTAAGGGGTGGT – (CH_2_)_7_ –NH– Methylene Blue – 3’

### Electrode fabrication and functionalization

To fabricate the electrodes of intravenous sensors, we cut and insulated gold (0.2 μm diameter × 10 cm in length; 99.9% purity, A-M systems), platinum (0.125 μm diameter × 10 cm in length; 99.95% purity; A-M Systems), and silver (0.125 μm diameter × 10 cm in length; 99.99% purity, A-M Systems) wires with polytetrafluoroethylene heat-shrink (PTFE, Zeus Inc., HS Sub-Lite-Wall). We bundled the wires with physical gaps separating each wire to prevent shorting. We then trimmed the insulation to produce an exposed length of 3 mm (gold), 5 mm (platinum), and 1 cm (silver). To convert the silver wire to a reference electrode we submerged it in 7.5% sodium hypochlorite (commercial bleach, Clorox) overnight to form a stable silver chloride film. Finally, we rinsed the electrodes in DI water to remove any residual bleach.

To fabricate the electrodes of subcutaneous sensors, we insulated gold (0.2 μm diameter × 10 cm in length), and silver wire (0.2 μm diameter, 10 cm in length, 99.99% purity, A-M Systems) with polytetrafluoroethylene heat-shrink (PTFE, HS Sub-Lite-Wall). We trimmed the insulation and electrodes to produce an exposed length of 5 mm (gold), and 1 cm (silver). To convert the silver wire to a reference electrode we submerged it in 7.5% sodium hypochlorite (commercial bleach, Clorox) overnight to form a stable silver chloride film, followed by a DI water wash. We then fabricated a separate counter electrode by heat-shrinking PTFE platinum wire (0.25 μm diameter × 10 cm in length). We then fed the counter through a 20-gauge catheter.

We functionalized intravenous and subcutaneous sensors as follows. First, we reduced the disulfide bond in the methylene-blue-modified DNA with 14 μL of 10 mM tris (2-carboxyethyl) phosphine with 2 μL of 100 mM DNA for 1 h in the dark. After electrode assembly and overnight bleaching we rinsed the sensors in di-ionized water and cleaned the gold surface via: (a) cycling the potential 1,000 times between − 1.0 and − 1.6 V versus Ag|AgCl in a solution of 0.5 M NaOH (1 Vs^− 1^) to remove residual organic or thiol contaminants on the surface; (b) pulsing between 0 and 2 V by applying 32,000 pulses with a pulse length of 20 ms in 0.5 M H_2_SO_4_ to increase the microscopic surface area of the gold.^38^ Following this, we rinsed the gold electrodes in de-ionized water, fed them through 20 (intravenous) and 22 (subcutaneous)-gauge catheters, and immersed them in the reduced DNA solution (500 nM in pH = 7.4 PBS) for 1 h. The gold electrodes were then transferred to a 10 mM solution of 6-mercapto-1-hexanol in PBS overnight at room temperature to complete the formation of their self-assembled monolayers. Finally, we fed the intravenous and subcutaneous sensors and the external counter of the latter through the lumen of 22 and 20-gauge catheters, respectively (Becton, Dickinson & Company). Before in vivo insertion, we fill these catheters with 1x PBS.

We performed our in vivo experiments using adult male Sprague-Dawley rats (4–5 months old, 400–650 g; Charles River Laboratories, Wilmington, MA, USA). These were pair-housed in a temperature and humidity-controlled vivarium on a 12-h light-dark cycle and provided ad libitum access to food and water. All animal procedures were consistent with the guidelines of the NIH Guide for Care and Use of Laboratory Animals (8th edition, National Academy Press, 2011) and approved by the Institutional Animal Care and Use Committee (IACUC) of the University of California Santa Barbara.

We performed sensor placements as follows. Rats were induced under 4% isoflurane gas in a Plexiglas anesthesia chamber. Anesthesia was maintained with 2–3% isoflurane gas/oxygen administered via a nose cone for the experiment’s duration. A pulse oximeter (Nonin Medical, Plymouth, MN) was used to measure heart rate and SpO2 during the experiment. The rat was shaved and the skin above the jugular vein was disinfected with 70% ethanol and betadine. A small incision was made to isolate both veins. A small incision in the jugular vein was made using spring-loaded microscissors. A silastic catheter (composed of a bent steel cannula and silastic tubing) was inserted into the left jugular vein for infusions. The sensor was inserted into the right jugular vein for in-vein drug monitoring and stabilized with sterile 6 – 0 silk sutures (Fine Science Tools, Foster City, CA). Following this insertion, we infused 30 units of heparin through the indwelling infusion line. Insertion of the subcutaneous sensor and external counter electrode was performed using an 18 g catheter inserted just below the surface of the skin on the posterior ventral side of the rat between the two legs after shaving the area. We confirmed the guide catheter is not embedded in fat or muscle by touch (the latter offer significant resistance to insertion). To ensure that we sampled ISF and not blood, we confirmed that the catheter did not backfill with blood during initial insertion and checked to ensure the sensor was free of blood when it was removed after the experiment. Neither of these occurred during the collection of any of the data presented in this study.

Sensor interrogation was performed electrochemically using square-wave voltammetry on a CH1040C multipotentiostat. To determine the relevant calibration curve, we performed a 24-point titration of each aptamer deposited onto the electrodes described above in 37° bovine blood and determined the “signal-on” (200 Hz) and “signal-off” (40 Hz) frequencies that resulted in the largest relative changes in current. Drift correct was performed with kinetic differential measurements (KDM), obtained by taking the difference in normalized peak currents collected at our signal-on and signal-off frequencies:

Eq. 1. $$KDM=\frac{\left({\text{Signal}}_{\text{on}}-{\text{Signal}}_{\text{off}}\right)}{\frac{1}{2}\left({\text{Signal}}_{\text{on}}+{\text{Signal}}_{\text{off}}\right)}$$

To fit resulting KDM signals to drug concentrations, we fit in vitro titration data to the equation:

Eq. 2. $$KDM={KDM}_{min}+\frac{{\left({KDM}_{max}-{KDM}_{min}\right)}^{*}[\text{Target}{]}^{n}H}{[\text{Target}{]}^{n}H+{K_{d}}^{n}H}$$

where ${KDM}_{max}$ is the maximum signal gain observed at saturating concentrations, [Target] is the drug concentration, $n_{H}$ is the Hill Coefficient, and $K_{D}$ is the binding half-point of the aptamer.^15^

### Feedback control

To perform feedback control, we employed an adaptive controller we have recently described.^32^ After a 20-minute baseline was measured to establish sensor stability, we started the adaptive feedback control algorithm. This algorithm does not use any a priori knowledge of the pharmacokinetics of doxorubicin in the test subject or any other animal. Instead, it uses a simple, pre-defined, time-varying infusion rate until the drug level reaches 65% of the desired set point. Using the injection profile and the resulting plasma concentrations measured up to this point, the control algorithm then fits the individual animal’s pharmacokinetics to a two-compartmental pharmacokinetic model. Afterward, the controller continues to update this pharmacokinetic model using the most recent 100 concentration measurements. This allows it to adapt to any changes in the animal’s physiology during the experiment. Using this model, the controller then controls the infusion rate to achieve and maintain the set point concentration. The controller also includes a safety feature that halts drug infusion if the drug concentration exceeds 120% of the set point concentration and turns back on it once the concentration falls back below 110% of the set point.

## Figures and Tables

**Table 1:** Rise times in plasma and ISF

Rat	Rise time in the plasma* (min)	Rise time in the ISF** (min)
Rat 1	6	25
Rat 2	11	29
Rat 3	10	59
Rat 4	5	SubQ1: 59 SubQ1: 37
Rat 5	2	SubQ1: 35 SubQ2: 34
**Average**	**6.8**	**39.7**

* Rise time (vein) is defined as the time elapsed between the initiation of control and when the plasma concentration reaches 15% of the set point concentration

** Rise time ISF is defined as the time elapsed between the initiation of control and when the subcutaneous concentration reaches 15% of the set point concentration

**Table 2:** The correlation between measured plasma and ISF concentrations

Rat identifier	Average and standard deviation of plasma concentration after the 2 μM set point is first reached (μM)	Average and standard deviation of subcutaneous concentration after the 2 μM set point is first reached (μM)
Rat 1	2.2 ± 0.4	2.0 ± 0.1
Rat 2	1.9 ± 0.2	2.10 ± 0.07
Rat 3	2.2 ± 0.3	2.2 ± 0.3
Rat 4	2.0 ± 0.4	SubQ1: 1.8± 0.1 SubQ2: 1.9± 0.1
Rat 5	2.0 ± 0.2	SubQ1: 1.80 ± 0.09 SubQ2: 1.90 ± 0.14

Average plasma and subcutaneous drug concentrations observed after the set point is reached (i.e., after the subcutaneous concentration reaches and remains for 5 min within 15% of the set point).

## Data Availability

All data needed to evaluate the conclusions in the paper are present in the paper and/or the Supplementary Materials.
